# An Aggregation-Induced Fluorescence Probe for Detection H_2_S and Its Application in Cell Imaging

**DOI:** 10.3390/molecules29102386

**Published:** 2024-05-19

**Authors:** Xin-Hui Tang, Hao-Na Zhang, Wen-Ling Wang, Qing-Ming Wang

**Affiliations:** School of Pharmacy, Yancheng Teachers University, Yancheng 224051, China; zhanghaonana2004@163.com (H.-N.Z.); wangwl@yctu.edu.cn (W.-L.W.)

**Keywords:** aggregation-induced emission, fluorescent probe, hydrogen sulfide, cell imaging

## Abstract

Monitoring hydrogen sulfide (H_2_S) in living organisms is very important because H_2_S acts as a regulator in many physiological and pathological processes. Upregulation of endogenous H_2_S concentration has been shown to be closely related to the occurrence and development of tumors, atherosclerosis, neurodegenerative diseases and diabetes. Herin, a novel fluorescent probe **HND** with aggregation-induced emission was designed. Impressively, **HND** exhibited a high selectivity, fast response (1 min) and low detection limit (0.61 μM) for H_2_S in PBS buffer (10 mM, pH = 7.42). Moreover, the reaction mechanism between **HND** and H_2_S was conducted by Job’s plot, HR-MS, and DFT. In particular, **HND** was successfully employed to detect H_2_S in HeLa cells.

## 1. Introduction

H_2_S is considered to be an important endogenous gaseous transmitter and plays a crucial role in the regulation of intracellular redox status, angiogenesis and anti-inflammatory effects in living organisms [1,2,3]. It is generated from L-cysteine or catalyzed by cysteine sulfide β-synthases and cysteine sulfide γ-lyase [4]. Evidence showed that the upregulation of H_2_S concentration is closely related to the occurrence and development of tumors, atherosclerosis, neurodegenerative diseases, diabetes and so on [5,6,7,8,9]. Therefore, it is necessary to construct a new H_2_S instantaneous detection method with high sensitivity and selectivity to evaluate H_2_S levels in biological systems.

Currently, many H_2_S-activatable small-molecule fluorescent probes have been developed based on different reaction mechanisms, such as the removal of copper ion via copper sulfide precipitation, nucleophilic addition or the reduction of azides/or nitros to amines mediated by the thiolysis of dinitrophenyl ether [7,10,11,12,13,14,15]. However, some of them are based on hydrophobic organic fluorescent groups, which have the disadvantage of poor water solubility and easy aggregation to form precipitates in aqueous solutions, thereby reducing the responsivity to the analyte and limiting the practical application of organic fluorescent molecular probes in biological imaging. Due to the advantages of excellent photostability and biocompatibility of aggregation-induced emission (AIE), a lot of fluorescent probes with AIE effect for H_2_S have been synthesized. However, most of the fluorescent probes with AIE effect are based on the molecular skeleton of tetrastyrene (TPE) [16,17] and have relatively complex molecular structures, which increases the difficulty of AIE molecular design and synthesis. Therefore, the design of efficient AIE bioluminescent materials with simple molecular structures and simple synthesis remains a challenge.

In this paper, a novel AIE activity fluorescent probe, named 2-hydroxy-1-naphthalaldehyde N-(dimethylamino)benzaldehyde (**HND**) was conducted. The mechanism was tested by Job’s plot, HR-MS, and DFT, and revealed that the two C=N double bonds in the structure of **HND** were used to provide a receptor unit for H_2_S. Compared with early reports (Table S1), **HND** exhibits a comparable limit of detection and a fast response. Furthermore, **HND** realized the detection of H_2_S in HeLa cells.

## 2. Results and Discussion

### 2.1. AIE Effect of HND in Solution

**Figure 1:**
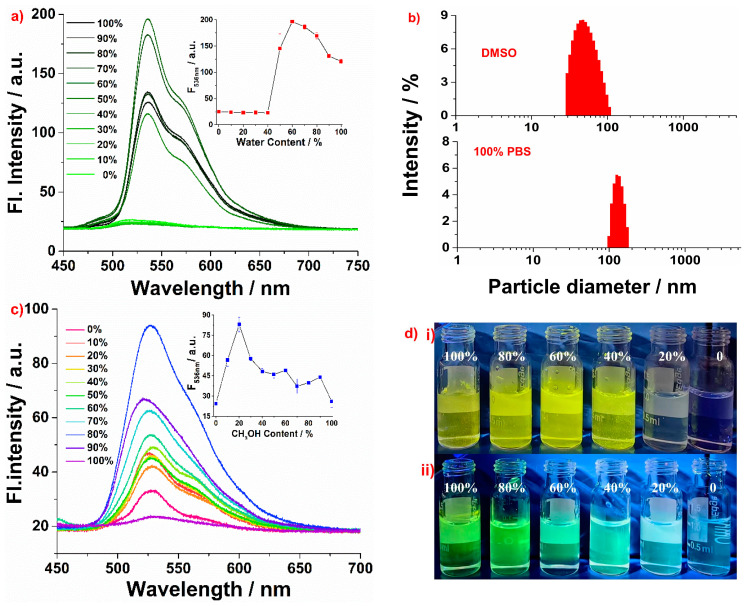
(**a**) Fluorescence spectra of **HND** (5.0 μM) in DMSO/water mixtures with different water fractions, pH 7.4. (**b**) Particle size analysis of **HND** in DMSO and PBS. (**c**) Fluorescence spectra of **HND** (5.0 μM) in CH_3_OH/glycerin mixtures with different glycerin fractions, λ_ex_ = 420 nm. (**d**) The corresponding fluorescence images of **HND** under 365 nm UV lamp illumination ((**i**) different PBS/DMSO mixtures; (**ii**) different CH_3_OH/glycerin mixtures).

To better understand the AIE effect of **HND**, the fluorescence properties of **HND** in a mixed solvent system of DMSO and H_2_O were first investigated. As an exception, **HND** showed no fluorescence signal when dissolved in the good solvent DMSO, while the fluorescence signal at 536 nm could be turned on and gradually increased with the increase in water volume fraction; an 8.23-fold increase was observed when the water volume fraction increased to 60% (as shown in Figure 1a). With the water volume fraction increased from 60% to 100%, the fluorescence signal reduced slightly. The photographs given in Figure 1d show the color change in **HND** with increasing water content. A clear yellow fluorescence was observed under a 365 nm lamp. These results indicated that the compound **HND** is a typical aggregation-induced emission (AIE) active compound in the aqueous medium [18]. Herein, DMSO is a good solvent and water is a poor solvent for **HND** [19]. The reason for the fluorescence enhancement of **HND** in DMSO is because the radiation channel was opened, leading to an increase in fluorescence intensity due to the restricted intramolecular rotations (RIRs) of the coumarin matrix and thiosemicarbazide [20].

Particle size analysis was a useful method to further verify the formation of aggregates of **HND**. As shown in Figure 1b, the average particle size of **HND** (5.0 μM) in DMSO solution was 45.69 nm, while the average diameter in PBS (10 mM, pH = 7.40) was approximately 128.52 nm, indicating that **HND** does indeed have aggregates in aqueous solutions. Furthermore, the diameter of **HND** was supplemented by transmission electron microscopy (TEM). The TEM results showed that the particle size of **HND** is approximately 100 nm, which is consistent with the results of particle size analysis (128.52 nm). In addition, TEM showed that the morphology of **HND** was almost elliptical and it had a structural feature with a darker color in the center and a thin film coating at the periphery (Figure S4).

To better understand the properties of AIE, we conducted analyses of the fluorescence spectra of **HND** (5.0 μM) in different CH_3_OH/glycerin mixtures. As shown in Figure 1c, the fluorescence intensity at 536 nm increased with the increase in glycerol fractions. The results indicated that the sticky glycerin could block the nonradiative intramolecular rotation [18]. This means that **HND** is a typical AIE compound [13,19].

### 2.2. Selectivity and Competition

**Figure 2:**
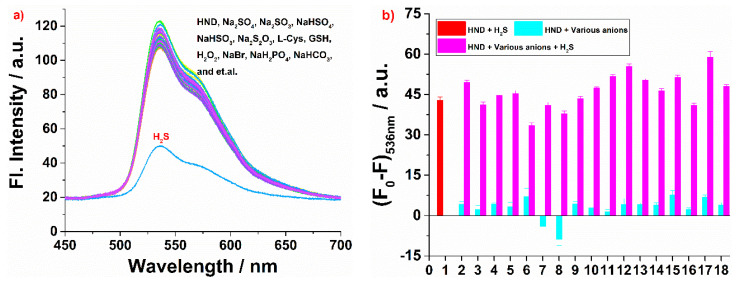
(**a**) Selectivity profile of 5.0 μM **HND** toward the RSS, various anions and H_2_O_2_ (500 μM) in PBS buffer (10 mM, pH 7.4), λ_ex_ = 420 nm. (**b**) Fluorescence intensity changes of 5.0 μM **HND** upon addition of 100 equiv. H_2_S and 100 equiv. of the RSS, various anions and H_2_O_2_, λ_em_ = 536 nm (1–18: **HND**, Na_2_S_2_O_3_, NaHSO_3_, Na_2_SO_4_, Na_2_SO_3_, L-Cys, GSH, H_2_O_2_, Na_3_PO_4_, NaI, NaF, NaCl, NaBr, Na_2_CO_3_, NaHCO_3_, Na_2_C_2_O_4_, NaNO_3_, NaNO_2_).

Many H_2_S probes based on nucleophilic addition reaction have been reported in recent years [15,21,22,23,24]. To identify the interaction between **HND** and H_2_S (Na_2_S was employed as H_2_S donor), as shown in Figure 2a, an extremely strong yellow fluorescence with λ_max_ = 536 nm was observed in PBS buffer solution (10 mM, pH = 7.40). However, the fluorescence intensity of **HND** (5.0 μM) was dramatically reduced after the addition of 100 eq. H_2_S in PBS buffer solution (10 mM, pH = 7.40). The fluorescence intensity of **HND** was decreased to almost no fluorescence. There are other biologically relevant reactive sulfurs (RSS), such as Na_2_SO_4_, Na_2_SO_3_, NaHSO_4_, NaHSO_3_, Na_2_S_2_O_3_, L-Cys and GSH, which could react with **HND**; here, it is necessary to verify the specificity of **HND**. In addition, many anions and cations, such as PO_4_^3−^, HCO_3_^−^, NO_2_^−^, Br^−^, NO_3_^−^, F^−^, CO_3_^2−^, Cl^−^, C_2_O_4_^2−^, I^−^, etc., were also investigated. The results indicated that no detectable responses were observed when 100 eq. of other RSS or many anions were added to **HND**, respectively. The response of **HND** to H_2_O_2_ was also conducted; it was found that H_2_O_2_ could not respond to **HND**.

In addition, a lot of metal ions, such as Mn^2+^, Cu^2+^, Ag^+^, Co^3+^, Fe^3+^, Al^3+^, Ca^2+^, K^+^, Ni^2+^, Cd^2+^, Cr^3+^ and so on, which coexist in the human body, were also investigated; except for Cu^2+^, all other tested metal ions have no interference with **HND** (Figures S5 and S6). All of the results showed that **HND** has high selectivity for H_2_S.

To further demonstrate the ability of **HND** to respond to H_2_S in the presence of other biologically relevant reactive RSS, anions and H_2_O_2_, the competition of **HND** was also examined (Figure 2b). It was found that **HND** could also respond to H_2_S in the presence of other biologically relevant reactive RSS, anions and H_2_O_2_, and the fluorescence intensity was similar to that of **HND** directly responding to H_2_S. This informed us that the RSS, anions and H_2_O_2_ tested had a negligible interfering effect on the detection of H_2_S by **HND**. Similarly, when metal ions and H_2_S coexisted, the decrease in fluorescence intensity of HND was also observed (Figure S6).

### 2.3. H_2_S Response by Probe HND

**Figure 3:**
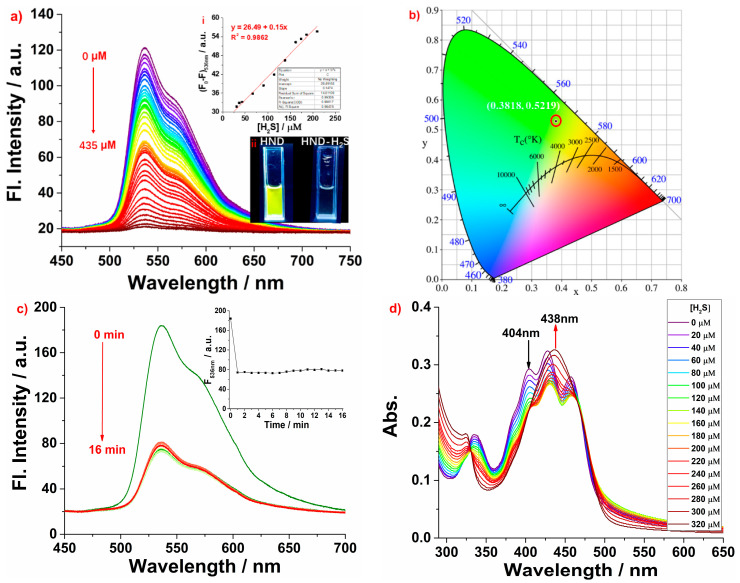
(**a**) The fluorescence titration curve of 5.0 μM **HND** in the presence of different concentrations of H_2_S. Inset: (**i**) The corresponding fluorescence images of **HND** and **HND** mixed with H_2_S under 365 nm UV lamp illumination. (**ii**) Changes in emission intensity at 536 nm with incremental additions of H_2_S. (**b**) CIE diagram of the **HND** in PBS buffer solution (10 mM, pH = 7.40). (**c**) Changes in emission intensity of **HND** (5.0 μM) at 536 nm in the presence of 10 equiv. of H_2_S as the function of time. (**d**) Absorption spectra of **HND** (15 μM) treated with different concentrations of H_2_S.

Figure 3a shows the relationship between **HND** and the concentrations of H_2_S. The fluorescence intensity of **HND** in PBS buffer solution (10 mM, pH = 7.40) at 536 nm decreased with the gradual addition of H_2_S at different concentrations. When 435 μM H_2_S was added, a 5.71-fold quenching of the fluorescence was observed. The fluorescence color changed from light yellow to colorless (shown in Figure 3a inset). A good linear relationship was observed between the fluorescence intensities at 536 nm, as well as the H_2_S concentration from 25 to 210 μM; moreover, the value of the linear correlation R^2^ was 0.9862 (Figure 3a inset). Followed by the equation LOD = K × Sb1/S [25], the detection limit for H_2_S was estimated to be 0.61 μM. Compared with the early results (as shown in Table S1), the LOD value is consistent with the reports by Lu and Jose [26,27,28] and good compared with the reports by Zhang [29,30]. Additionally, the pH-dependent responses of probe **HND** and **HND** to H_2_S were carried out. From Figure S7, it can be seen that probe **HND** could only respond to H_2_S at pH 7.42. At other pH values, such as 4.00, 6.86, 9.18 and 10.01, **HND** could not respond to H_2_S. Therefore, PBS buffer (10 mM, pH 7.4) was chosen as the reaction system.

CIE chromaticity was used to better understand the chromaticity changes in the fluorescence spectra of **HND** upon the addition of H_2_S [31]. CIE chromaticity coordinates were also calculated from the emission spectrum [32]. A two-dimensional space (XY plane) could be found in the CIE where each point of the chromaticity map stands for a particular color. From Figure 3b, the CIE chromaticity of **HND** was found to be x = 0.3818, y = 0.5219, while after the addition of H_2_S, the CIE chromaticity was converted to be x = 0.3618, y = 0.4174. This is an indication of a gradual shift in the color (Figure S8).

Response time is one of the most important parameters for a sensor. Therefore, kinetic studies were conducted to investigate the response time. A total of 10 eqiv. of H_2_S was added to **HND** (5.0 μM) and the fluorescence intensity at 536 nm was monitored over time until the spectrum became constant (Figure 3c). The results showed that it takes almost 1 min for **HND** to react completely with H_2_S. This is a fast reaction rate compared to the first report [13,28,33,34].

Furthermore, the absorption spectra of **HND** (15 μM) treated with different concentrations of H_2_S in PBS buffer solution (10 mM, pH = 7.40) were recorded. As shown in Figure 3d, four peaks at 457 nm, 427 nm, 404 nm and 355 nm were observed; with the addition of H_2_S, the peaks at 457 nm, 427 nm, 404 nm and 355 nm were decreased and two new peaks at 438 nm and 326 nm appeared and gradually increased. An isoabsorption point at 329 nm was found. The isoabsorptive point indicated that a new compound was formed between **HND** and H_2_S [31,35].

### 2.4. Mechanism Investigation

**Figure 4:**
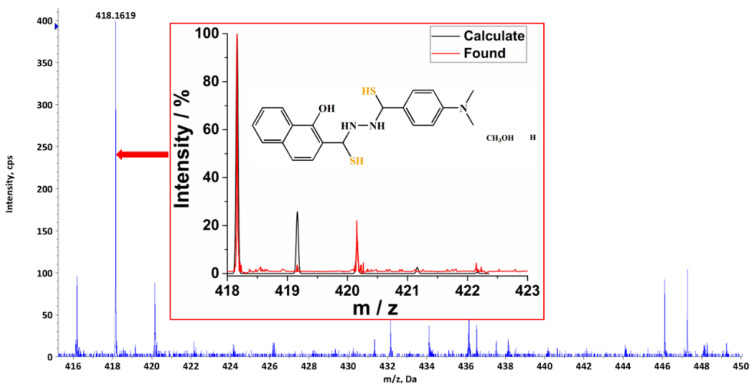
ESI mass spectrum of **HND** upon addition of excess Na_2_S.

HR-MS was used to further investigate the mechanism involved in the fluorescence quenching between **HND** and Na_2_S. The HR-MS of **HND** shows a strong peak at *m*/*z* = 318.1606 [**HND** + H] ^+^ (Figure S2). After the addition of an excess of Na_2_S, a new peak appears at *m*/*z* = 418.1619 [**HND** -HS + CH_3_OH + H]^+^, which indicates clearly that the C=N of **HND** were formed through nucleophilic addition by H_2_S in aqueous solution (Figure 4). The recognition mechanism is similar to the other reports [36,37,38,39].

**Figure 5:**
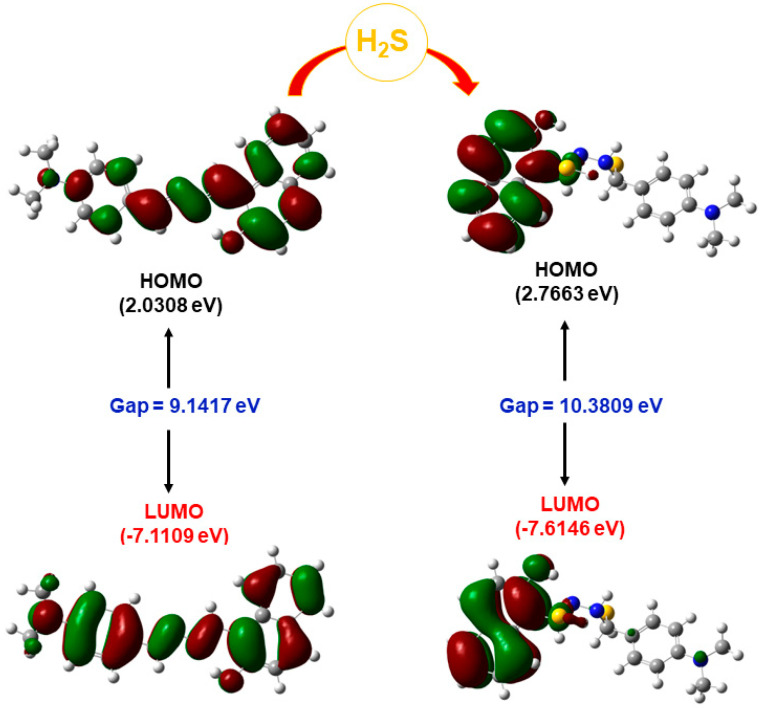
HOMOs and LUMOs of **HND** and **HND** -HS.

DFT with B3LYP/6-31G(d) (the Gaussian 09) was carried out to further investigate the electron distributions and orbital energies of **HND** and H_2_S [40,41]. From Figure 5, we find that the highest occupied molecular orbitals (HOMOs) and the lowest occupied molecular orbitals (LOMOs) of **HND** were mainly distributed over the whole molecule, so **HND** showed a strong fluorescence due to the absence of electron transfer. The HOMOs and LUMOs of **HND** -HS were mainly distributed on the 2-hydroxy-1-naphthylaldehyde group. The HOMO-LUMO energy gaps of **HND** and **HND** -HS were 9.1417 ev and 10.3809 ev, respectively. The HOMO-LUMO band gap of **HND** -HS was higher than that of **HND**, which could be clearly explained by the blue shift in the emission maxima of **HND** -HS [32,42].

### 2.5. Fluorescence Imaging

**Figure 6:**
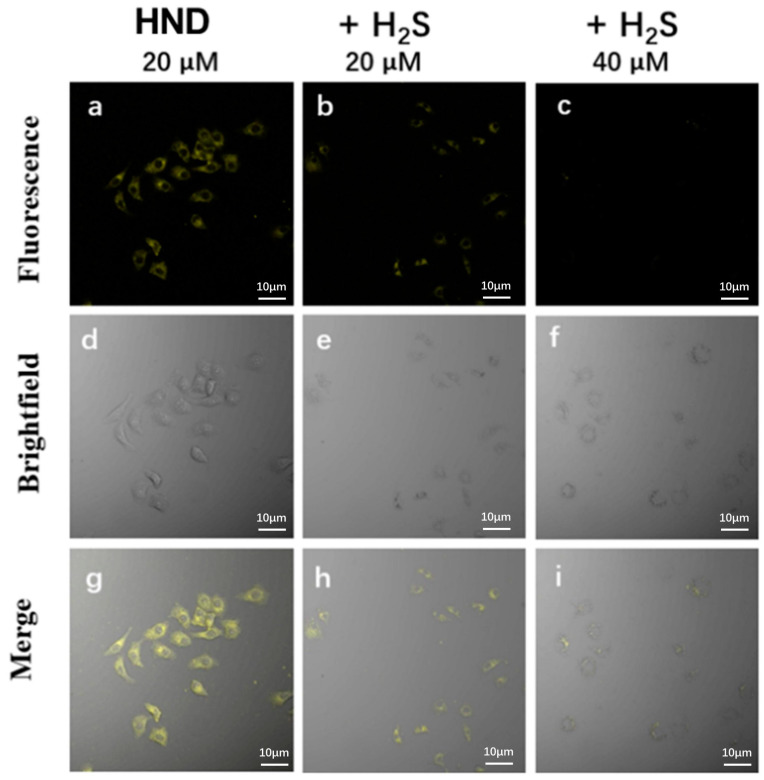
Fluorescence microscopy images of HeLa cells. (**a**,**d**,**g**) HeLa cells incubated with **HND** (20 μM) for 20 min. (**b**,**e**,**h**) HeLa cells pretreated with **HND** (20 μM) and then treated with H_2_S (20 μM) for 20 min. (**c**,**f**,**i**) HeLa cells pretreated with **HND** (20 μM) and then treated with H_2_S (40 μM) for 20 min.

The applicability of **HND** in biological systems was investigated by analyzing H_2_S imaging in living cells. Bright yellow fluorescence was obtained when HeLa cells were incubated with 20 μM of **HND** for 20 min. However, the bright yellow fluorescence of HeLa cells was gradually quenched when the cells were treated with **HND** for 20 min and then treated with H_2_S (the concentrations of H_2_S were 20 μM and 40 μM, respectively) for another 20 min (Figure 6b,c). The results indicated that **HND** has good biocompatibility and is able to image different concentrations of H_2_S in living cells.

## 3. Materials and Methods

### 3.1. Materials

All reagents were purchased through a commercial method and used without further purification. ^1^H-NMR was obtained using a Bruker DRX 400 spectrometer with TMS as the internal standard. High-resolution mass spectrometry (HR-MS) was performed on a Triple TOF TM 5600^+^ system. Fluorescence spectra were obtained at room temperature using a CRT-970 fluorescence spectrophotometer. The absorption spectra were measured by Shimadzu UV1800. Imaging of probe **HND** and **HND** with Na_2_S was conducted on a confocal laser scanning microscope L700 with HeLa cells.

### 3.2. The Synthesis Process

**Scheme 1:**
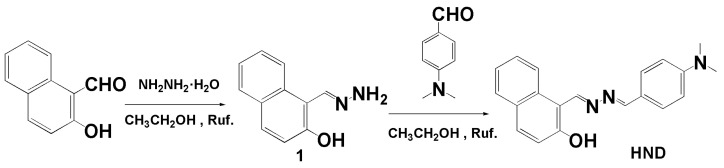
Design and synthesis of the **HND**.

As shown in Scheme 1, first, the mixture of ethanol (30 mL), 2-hydroxynaphthalene-1-carbaldehyde (0.344 g, 2.0 mmol) and hydrazine hydrate (400 μL) were first refluxed in a flask. After 10 h, the reaction was stopped by TLC. Yellow solids were obtained, named 2-hydroxynaphthalene-1-carbaldehyde hydrazone (**1**). Next, 1 (0.279 g, 1.5 mmol) and 4-(dimethylamino)benzaldehyde (0.1491 g, 1.0 mmol) were mixed in 30 mL ethanol, a few drops of DMF was added as a catalyst, and then the above mixture was heated and refluxed for 24 h. The yellow powder **HND** was obtained after the mixture was cooled and washed by ethanol three times; then, it was recrystallized by CH_3_CH_2_OH. Yield, 0.17 g (54%). ^1^H-NMR (400 MHz, DMSO-*d_6_*) *δ* 12.90 (s, 1H), 10.01 (s, 1H), 8.66 (d, J = 8.6 Hz, 1H), 8.05 (d, J = 8.9 Hz, 1H), 7.93 (d, J = 7.3 Hz, 1H), 7.63 (t, J = 7.7 Hz, 1H), 7.45 (t, J = 7.1 Hz, 1H), 7.29 (d, J = 9.0 Hz, 1H), 1.23 (s, 6H) (Figure S1). HR-MS: *m*/*z* = 318.1606 [**HND** + H]^+^, [**HND** + H]^+^ found 318.1606 (Figure S2). **HND** showed the yellow fluorescence in the solid under 365 nm UV lamp illumination (Figure S3).

### 3.3. Fluorescence Spectral Property

Na_2_S was dissolved in water and used as the donor of H_2_S [36]. Stock solution of probe **HND** (10^−3^ M) was prepared in DMSO. The fluorescence spectra of HND with H_2_S were obtained by the addition of various concentrations of Na_2_S to **HND** (5.0 μM) in PBS buffer (10 mM, pH 7.4). The fluorescence spectra were excited by 420 nm.

## 4. Conclusions

In conclusion, a novel fluorescent probe **HND** with AIE activity was synthesized. The results showed that **HND** could be used for the detection of H_2_S in aqueous solution and HeLa cells. **HND** exhibited obvious fluorescence quenching and excellent selectivity/competitiveness toward H_2_S with a limit of detection of 0.64 μM and a response time of 1 min. We assumed that H_2_S reacted with **HND** through nucleophilic addition reaction and the mechanism was proven by HR-MS and DFT calculations. In addition, **HND** was successfully applied to image the H_2_S in HeLa cells.

## Data Availability

The original contributions presented in the study are included in the article/supplementary material, further inquiries can be directed to the corresponding authors.

## Supplementary Materials

The following supporting information can be downloaded at: https://www.mdpi.com/article/10.3390/molecules29102386/s1, Figure S1: ^1^H-NMR of **HND**. Figure S2: HR-MS of **HND**. Figure S3: The color of **HND** as a solid. (Right) under 365 nm UV lamp illumination; (left) under visible light. Figure S4: TEM of **HND** (5.0 μM) in 100% PBS. Figure S5: Selectivity profile of 5 μM **HND** toward various metal ions (500 μM) in PBS buffer (10 mM, pH 7.4), λ_ex_ = 420 nm. Figure S6: Fluorescence intensity changes of 5.0 μM **HND** upon addition of 100 equiv. H_2_S and 100 equiv. of various metal ions, λ_em_ = 536 nm. Figure S7: The pH-dependent responses of probe **HND** and **HND** to H_2_S. Figure S8: CIE diagram of the **HND -HS** in PBS buffer solution (10 mM, pH = 7.40). Figure S9: Time-dependent fluorescence intensity of **HND** (5.0 μM) in PBS (pH 7.4, 10 mM). Table S1: Comparison of **HND** for the detection of H_2_S. References [43,44,45] are cited in Supplementary Materials.
