# Supplementary material for: An Aggregation-Induced Fluorescence Probe for Detection H2S and Its Application in Cell Imaging
Source: Molecules. 2024 May 19;29(10):2386. doi: 10.3390/molecules29102386 (PMC11124099; doi:10.3390/molecules29102386)
Supplement: Supplementary file 1 [file molecules-29-02386-s001.zip › molecules-3007527-supplementary.pdf]

Supporting Information

An Aggregation-Induced Fluorescence Probe for Detection H_2S and Its Application in Cell Imaging

Xin-Hui Tang *, Hao-Na Zhang, Wen-Ling Wang and Qing-Ming Wang *

School of Pharmacy, Yancheng Teachers University, Yancheng 224051, China; zhanghaonana2004@163.com (H.-N.Z.); wangwl@yctu.edu.cn (W.-L.W.)

* Correspondence: xinhuitang@sina.com (X.-H.T.); wangqm@yctu.edu.cn (Q.-M.W.)

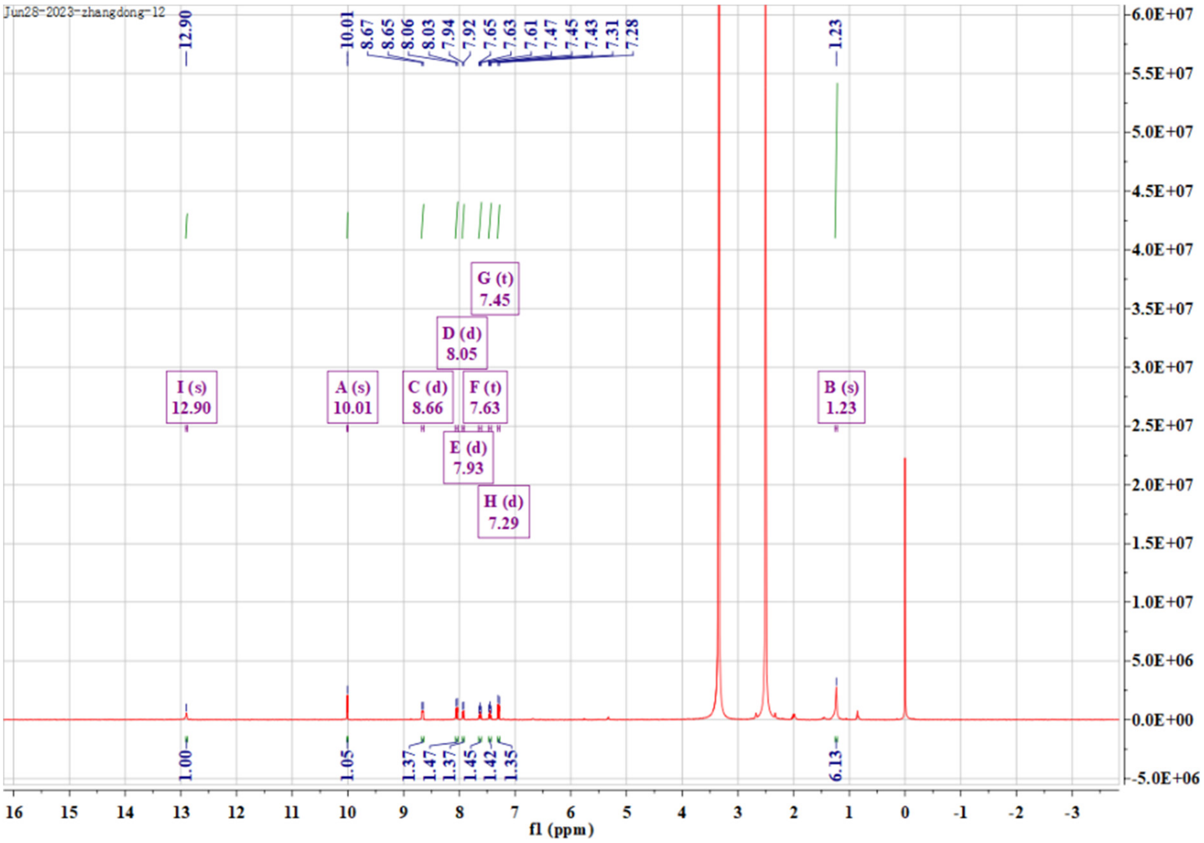

Figure S1. ^{1}H-NMR of **HND**.

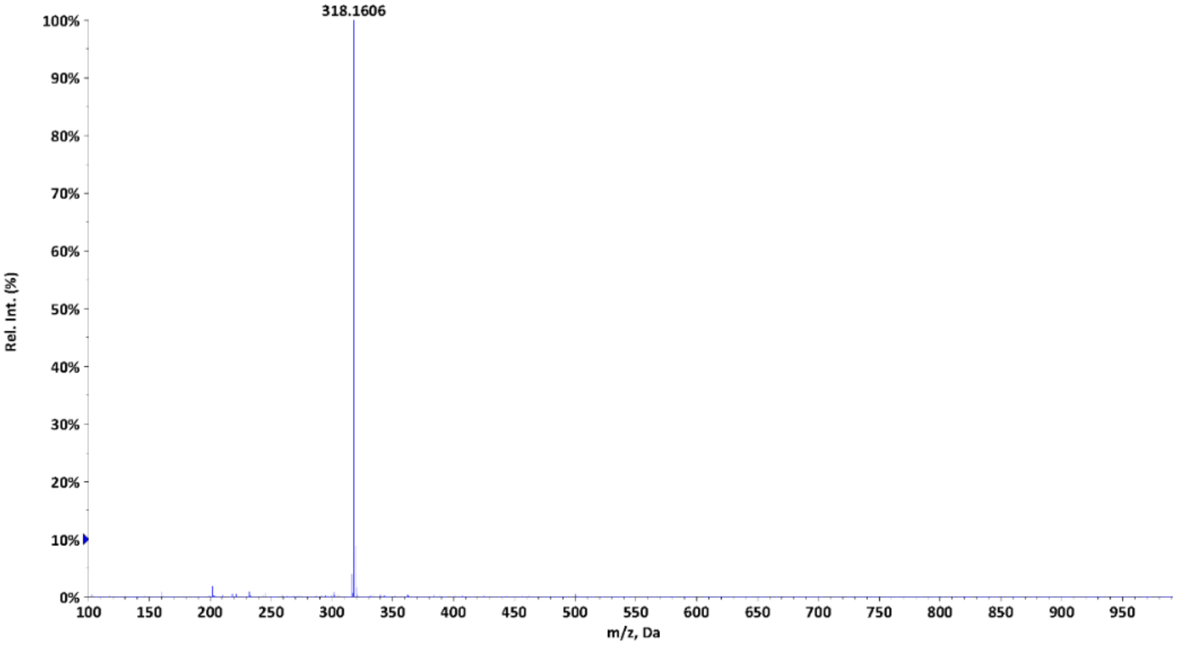

Figure S2. HR-MS of **HND**.

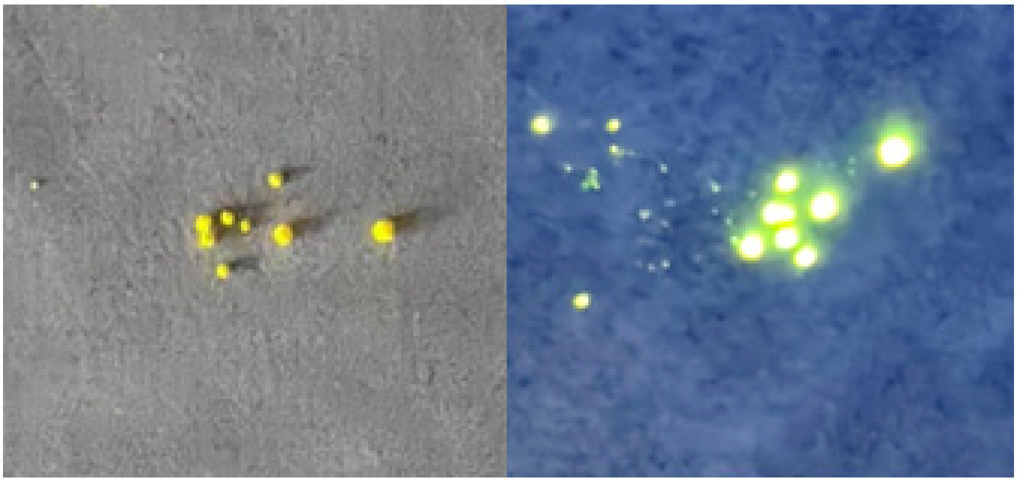

Figure S3. The color of **HND** in solid. (Right) under 365 nm UV lamp illumination; (Left) under Visible light.

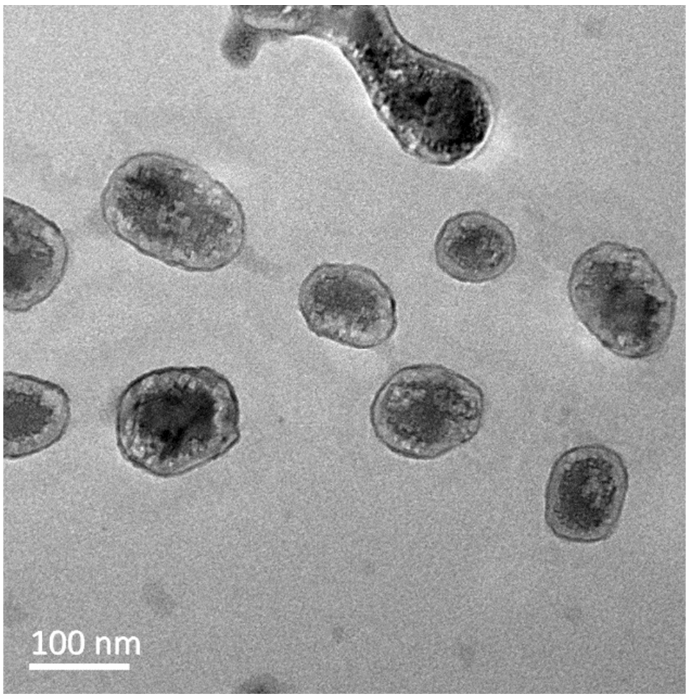

Figure S4. TEM of **HND** (5.0 μM) in 100% PBS.

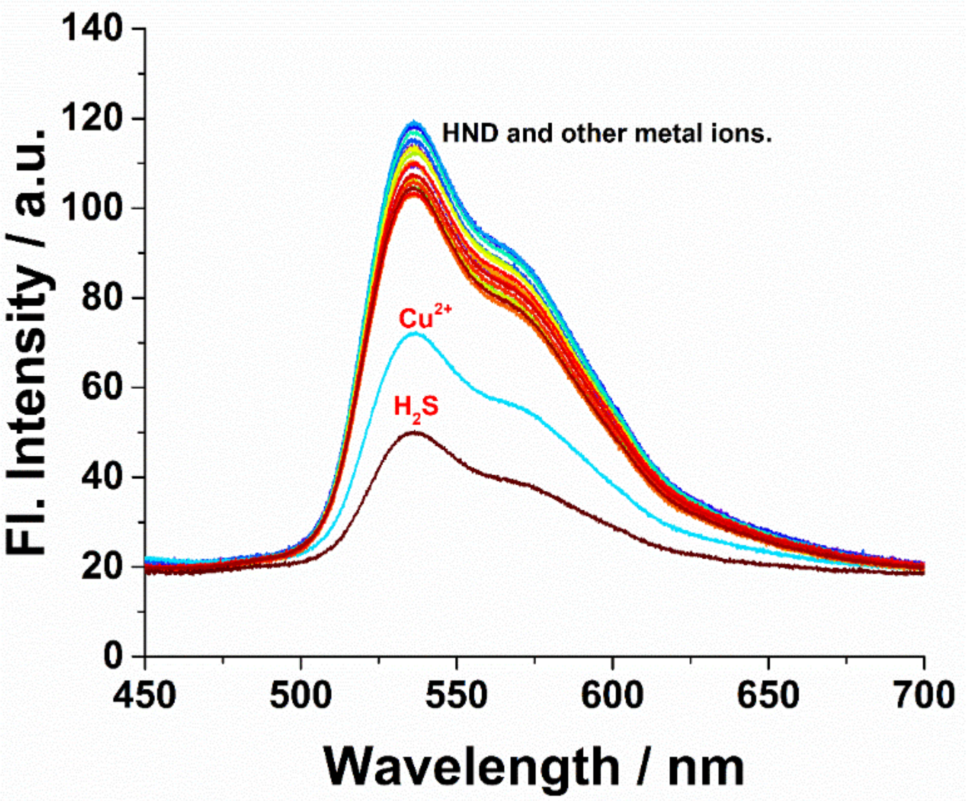

Figure S5. Selectivity profile of 5 μM **HND** toward various of metal ions (500 μM) in PBS buffer (10 mM, pH 7.4), λ_{ex} = 420 nm.

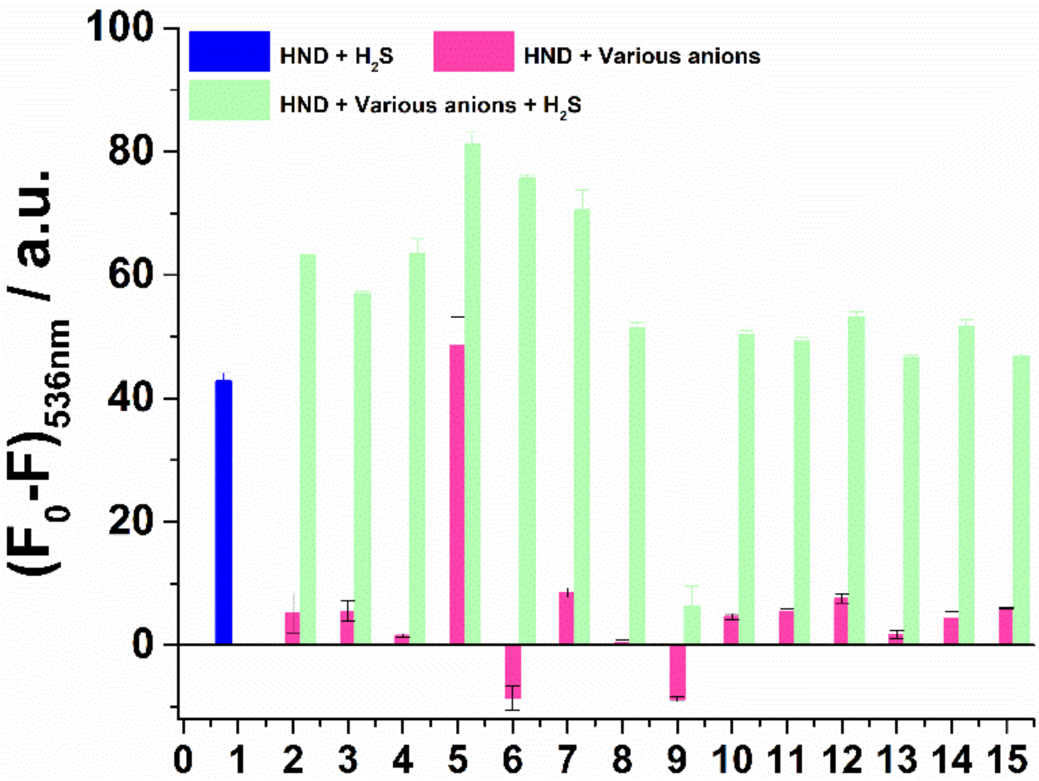

Figure S6. Fluorescence intensity changes of 5.0 μM **HND** upon addition of 100 equiv. H_2S and 100 equiv. various of metal ions, λ_{em} = 536 nm.

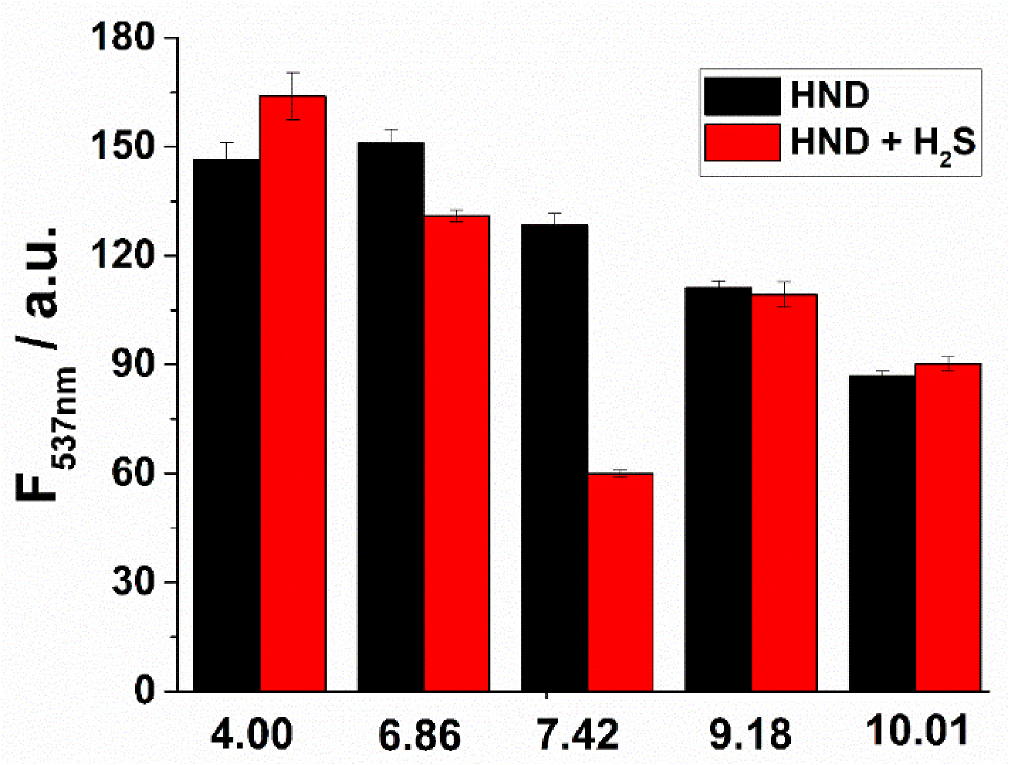

Figure S7. The pH-dependent responses of probe **HND** and **HND** to H_2S.

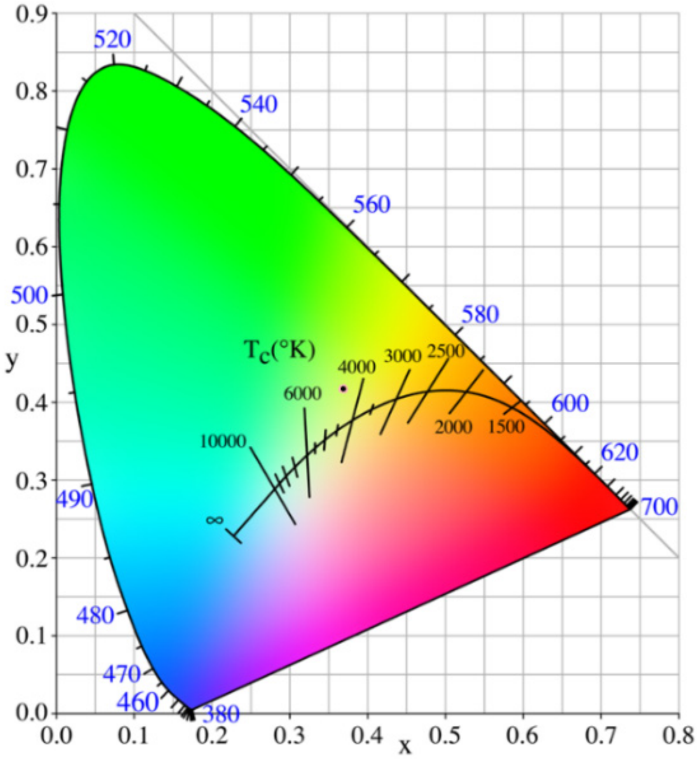

Figure S8. CIE diagram of the **HND-HS** in PBS buffer solution (10 mM, pH = 7.40).

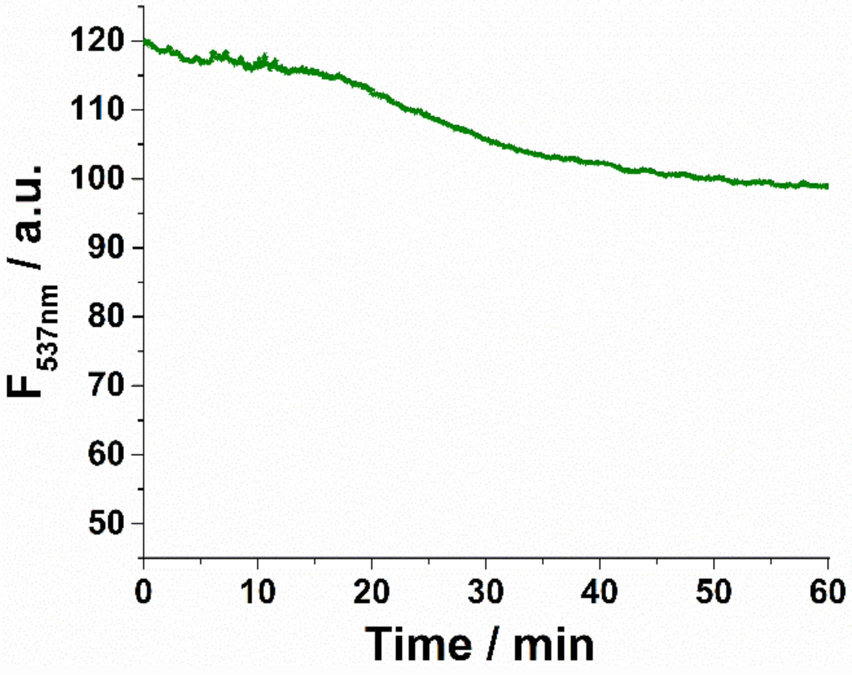

Figure S9. Time-dependent fluorescence intensity of **HND** (5.0 μM) in PBS(pH 7.4, 10mM).

Table S1. Comparison of **HND** for the detection of H_2S.

	Detection limit	Response time	Solvent	Applications	Ref.
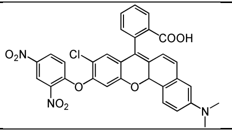	0.31 μM	2 min	PBS buffer (pH = 7.4)	Cell Imaging	R1
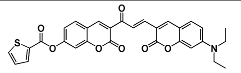	0.323 μM	30 s	(pH 7.4, including 30% DMSO as cosolvent)	Cell Imaging	R2
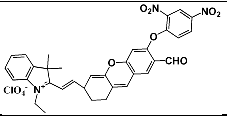	0.179 μM	150s	EtOH/PBS buffer	Hydrotalcite tablet samples	R3
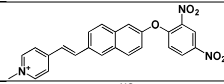	11.2nM	15 min	BR buffer solution (pH = 7.42	Cell imaging	R4
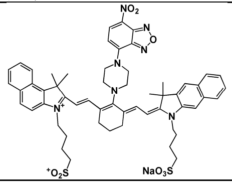	0.27 μM	-	PBS buffer (pH = 7.4)	Cell imaging	R5
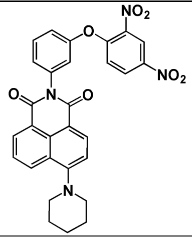	18.8nM	-	PBS buffer (H_2O/DMSO = 99:1, *v*/v)	Cell Imaging	R6
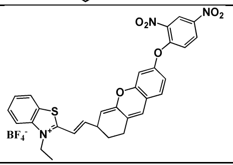	0.116 μM	120 min	PBS buffer (H_2O/C2H5OH = 4:1, *v*/v)	Cell imaging	R7
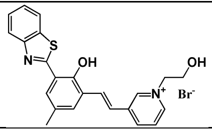	8.5 μM	30-60s	PBS/CH_3CN buffer (10 mM, pH = 7.4, 1/1, v/v)	Cell imaging	R8
This work	0.61 μM	1 min	PBS buffer (pH = 7.4)	Cell imaging	

R1. K. Wang, X. Yang, M.-Y. Guo, X.-Y. Chen, T. Li, R. Yan, Y.-S. Yang, H.-L. Zhu and Z.-G. Hu. Imaging the dynamic processes of hydrogen sulfide using a rapid "turn-on" mitochondria-targeting fluorescent probe. Sensors and Actuators B: Chemical, 2022, 369, 132285.

R2. X. Chen, D. He, X. Yang, F. Yuan, S. Yang, Y. Yang, K. Wang, J. Qian and L. Long. Construction of bifunctional fluorescent probe for two-step cascade recognition of hydrogen sulfide and biothiols in biological system. Sensors

and Actuators B: Chemical, 2023, 381, 133440.

R3. X. Qin, X. Liu, J. Wang, H. Chen and X. C. Shen. A NIR ratiometric fluorescent probe for the rapid detection of hydrogen sulfide in living cells and zebrafish. Talanta, 2024, 266, 125043.

R4. G. Chen, J. Xu, Z. Zhan, X. Gao, M. Jiao, M. Ren, L. Lv, T. Chen, Y. Li and Y. Liu. A bright two-photon fluorescence probe with large stokes shift for deep tissue imaging of H2S during metabolism. Dyes and Pigments, 2020, 172, 107850.

R5. J. Xiong, L. Xia, Q. Huang, J. Huang, Y. Gu and P. Wang. Cyanine-based NIR fluorescent probe for monitoring H_2S and imaging in living cells and in vivo. Talanta, 2018, 184, 109-114.

R6. P. Ou, Y. Wang, C. Hao, Y. Peng and L. Y. Zhou. Naphthalimide-based a highly selective two-photon fluorescent probe for imaging of hydrogen sulfide in living cells and inflamed tissue of mouse model. Spectrochim Acta A Mol Biomol Spectrosc, 2021, 245, 118886.

R7. C. Liu, Q. Liu, S. Cai, H. Ding, S. He, L. Zhao, X. Zeng and J. Gong. Novel near-infrared spectroscopic probe for visualizing hydrogen sulfide in lysosomes. Spectrochim Acta A Mol Biomol Spectrosc, 2022, 271, 120917.

R8. Y. Du, H. Wang, T. Zhang, W. Wen, Z. Li, M. Bi and J. Liu. An ESIPT-based fluorescent probe with fast-response for detection of hydrogen sulfide in mitochondria. Spectrochim Acta A Mol Biomol Spectrosc, 2022, 265, 120390.
